# Automated software for counting and measuring *Hyalella* genus using artificial intelligence

**DOI:** 10.1007/s11356-023-30835-8

**Published:** 2023-11-22

**Authors:** Ludy Pineda-Alarcón, Maycol Zuluaga, Santiago Ruíz, David Fernandez Mc Cann, Fabio Vélez, Nestor Aguirre, Yarin Puerta, Julio Cañón

**Affiliations:** 1 Environmental Management and Modeling Group (GAIA), Environmental School, Engineer Faculty, Universidad de Antioquia, Medellín, Colombia; 2 Power Electronics, Automation, and Robotics Group (GEPAR), Engineer Electronic, Engineer Faculty, Universidad de Antioquia, Medellín, Colombia; 3 Limnology and Environmental Modeling Group (GEOLIMNA), Environmental School, Engineer Faculty, Universidad de Antioquia, Medellín, Colombia

**Keywords:** Measuring protocols, Morphological traits, Image capture, Macroinvertebrates, Deep learning

## Abstract

Amphipods belonging to the *Hyalella* genus are macroinvertebrates that inhabit aquatic environments. They are of particular interest in areas such as limnology and ecotoxicology, where data on the number of *Hyalella* individuals and their allometric measurements are used to assess the environmental dynamics of aquatic ecosystems. In this study, we introduce HyACS, a software tool that uses a model developed with the YOLOv3’s architecture to detect individuals, and digital image processing techniques to extract morphological metrics of the *Hyalella* genus. The software detects body metrics of length, arc length, maximum width, eccentricity, perimeter, and area of *Hyalella* individuals, using basic imaging capture equipment. The performance metrics indicate that the model developed can achieve high prediction levels, with an accuracy above 90% for the correct identification of individuals. It can perform up to four times faster than traditional visual counting methods and provide precise morphological measurements of *Hyalella* individuals, which may improve further studies of the species populations and enhance their use as bioindicators of water quality.

## Introduction

*Hyalella* (Smith, 1874) is a crustacean of the Amphipod order that belongs to the ecological group of freshwater aquatic macroinvertebrates (Correa-araneda and Contreras 2010). Amphipods are an important link in the trophic chain that connects primary producers with higher trophic levels, thus constituting key pieces in the energetic transfer as prey for several depredators. (Arfianti and Costello 2020). This benthic organism lives in association with submerged aquatic vegetation (Bastos-Pereira and Bueno 2016; Reis et al. 2020) and reacts to the presence of pollutants in aquatic ecosystems (Amiard-Triquet and Berthet 2015). Therefore, *Hyalella* can serve as an indicator species that responds to changes in the quality of water and sediments. The individual response of *Hyalella* can be used to assess potential damage caused by different environmental stressors (chemical or physical), while changes in their population dynamics are of interest for monitoring aquatic ecosystems (Correa-araneda and Contreras 2010). The *Hyalella* genus is used as a bioindicator of high organic matter contents in water (Posada et al. 2000). Likewise, within the group of aquatic macroinvertebrates, amphipods, and particularly *hyalellas*, due to their narrow range of tolerance, are sensitive to environmental changes. Therefore, studies that involving the periodic counting of *Hyalellas* can be used for toxicology monitoring to asses influence of pesticides on the population growth (Beristain-Castillo et al. 2023) and as a biological model in ecotoxicological studies, given their high sensitivity to heavy metals (Colla and César 2019). Likewise, *Hyalella’s* high population density is related to fluctuations in electrical conductivity, temperature, and low concentrations of dissolved oxygen (Jacobsen and Marín 2008). These conditions could generate changes in dominance, diversity, reproductive strategies, and their relationship with physical and chemical environmental variables (Roldán-Pérez 2016; Stepanian et al. 2020), which determine the species permanence and survival strategies as a function of the quality of their environment (Nnoli et al. 2019; Zipkin et al. 2017). Other research identifies changes in the distribution of organisms that can represent a threat to the species of *Hyalella* genus, depending on the number of individuals and morphological size modifications (Zepon et al. 2021). The quantification of *Hyalella* organisms also helps to identify space–time trends at biological, ecological, and environmental levels (Bastos et al. 2021). These metrics are important in the description of new species, as described in the work of Rocha Penoni et al. (2021) which requires measuring the body length for species identification within *Hyalella* genus.

Currently, computer vision and digital image processing techniques are playing a relevant role in ecology and environmental engineering studies. These techniques facilitate various practical tasks in insect research, that involve observation, quantification, classification, measurement, and other data that can be inferred from images (Weinstein 2018).

Some examples of the applications of these techniques in insects and aquatic invertebrates include the application of digital imaging processing techniques to count fruit flies (Yati and Dey 2011) which is a fast, automatic, and simple process with a low error rate (1 to 4%); automatically classifying benthic macroinvertebrates into fine-grained categories using convolutional neural networks (CNN) such as MatConv, which has produced a new benchmark database with 64 categories of macroinvertebrates (Raitoharju et al. 2018), and automatically measuring the density of the copepod *Acartia tonsa,* which enables monitoring of metrics such as hatching rate, mortality, development rate, and individual biomass (Alver et al. 2011).

Sorting, identification, morphological and biological metrics, and biomass estimation of terrestrial and aquatic organisms can be accomplished through image identification using machine and deep learning techniques. For example, machine learning and deep learning have been used for the automatic sorting and extraction of metrics such as body length and volume from 14 common terrestrial invertebrate specimens, with an average precision of 91.4% for all taxa (Wührl et al. 2022). Furthermore, studies about image-based identification of terrestrial invertebrates using machine and deep learning could lead to improved understanding of these organisms (Ärje et al. 2020).

Other examples of the use of images in aquatic organisms include the extraction of morphological descriptors from in situ images of the Arctic zooplankton community, which has allowed for a better understanding of ecological patterns in the Arctic melt zone (Vilgrain et al. 2021). In addition, the movement patterns of *Oryzias latipes* were identified and measured after treatment with diazinon, using two-dimensional fast Fourier transform to efficiently calculate the differences before and after treatment (Park et al. 2005); Finally, real-time image analysis using digital image processing by binary image sequence was used to determine the movements of *Daphnia magna* in response to copper effects (Untersteiner et al. 2003);

Some applications, for instance ImageJ, which have a user interface, provide an accurate and flexible method for automating the analysis of digital photographs of laboratory microcosms. These applications can detect, count, and measure organisms, such as collembola, ants, nematodes, and daphnias, moving on a fixed but heterogeneous substrate (Mallard et al. 2013) or detect changes in the color of *Ulva pertusa* macroalgae (Lee et al. 2020);

Image processing in ecology is being used at different scales and process. For example, it is used to extract the mass of migratory insects based on an ellipsoidal dispersion model using radar data (Kong et al. 2019). It is also used for the segmentation and counting of pollen grains in microscopic images (Johnsrud et al. 2013); as well as for the processing and classification of four groups of aquatic macroinvertebrates (*Thraulodes, Traverella, Anacroneuria, and Smicridea*) through digital images processing for recognition at the genus taxonomic level with a 97.1% success rate (Serna López et al. 2020). Additionally, image processing, computer vision, and machine learning techniques are used for the registration and classification of *Rhopalosiphum padi* aphids to assess and predict crop damage through the use of Aphid CV software (Lins et al. 2020).

While basic image processing technics can solve a wide variety of environmental problems, some cases require more advanced tools related to pattern recognition. One such tool is YOLO (You Only Look Once) (Redmon et al. 2016) which is used to identify and locate objects in an image. YOLO consists of a convolutional neural network (CNN) based on deep learning that allows detecting and tracking down objects in an image, facilitating the quantification and obtaining of multiple metrics in images related to biological samples. According to previous work, when comparing YOLO with other object detection tools, it is much faster than other similar systems, since it applies a single CNN for the dual purpose of object classification and localization (Chen et al. 2022), which makes it ideal for real-time applications. Furthermore, YOLO has a higher accuracy and also is able to detect smaller objects than other systems (Wang et al. 2018). This is particularly useful in biological applications, such as detecting objects in microscopic images. Overall, YOLO’s combination of speed, accuracy, and small object detection capabilities make it a top choice for many developers and researchers in the computer vision field. Additionally, YOLO outperforms other object detectors like Faster R-CNN in terms of accuracy, speed, efficiency, and it is simpler to construct and can be trained directly on full images (Joiya 2022).

These image analysis tools contribute to the improvement of techniques for both counting individuals and quantifying the body measurements of specimens in research related to ecology and water. For instance, Zhong et al. (2018) developed a fast and accurate method of capturing, detecting, and counting flying insects based on YOLO object detector and support vector machines (SVM). Similarly, Kvæstad et al. (2022) developed a method to make morphological measurements of larvae images of *Gadus morhua* fish using deep learning with mask R-CNN neural net architecture.

The study of *Hyalella* populations requires the collection, separation, counting, and measurement of the organisms (Bastos-Pereira and Bueno 2016). There are widely established sampling protocols for collecting *Hyalella* (Rice et al. 2012; Ntislidou et al. 2021). However, the traditional method of separation and counting is performed manually under a stereomicroscope, which is subject to human errors of perception and lack of expertise. Counting individuals is a time-consuming activity that increases the likelihood of errors, due to fatigue and visual illusions (Lins et al. 2020).

Given that extracting information from images is quick, objectively verifiable, and less prone to observational errors (Pech et al. 2004), it is worth investing efforts in development tools that complement and improve traditional methods of information extraction. Manoukis et al. (2019) suggests that using techniques based on digital image processing could improve the efficiency of counting and measuring body morphological parameters such as length, maximum thickness, eccentricity, arc length, perimeter, and area, of individuals from the *Hyalella* genus. In this study, we are presenting the software HyACS (*Hyalella* Automatic Counting System), which is a form to call a tool developed to assist and enhance the work involving Hyalella and is aimed at detecting, counting, and extracting body metrics of the *Hyalella* genus individuals through deep learning and digital image processing techniques. This tool serves as an aid for researchers involved in aquatic ecology and water quality monitoring and offers advantages in efficiently and accurately obtaining data about metrics and counting *Hylella* organism. It allows researchers to automate the primary task related to this organism, which is quantifying the number of individuals with 92% of accuracy. Avoiding this task would rely on human observation, which can introduce errors. It allows for automatic analysis of a large set of individual image data, providing a novel approach for understanding both interspecific and intraspecific variability. Currently there is no other tool specifically designed for *Hyalella* organisms. Although there are similar tools, they are developed for other organisms, such as Aphid CV software for terrestrial aphids (Lins et al. 2020).

## Materials and methods

**Fig. 1:**
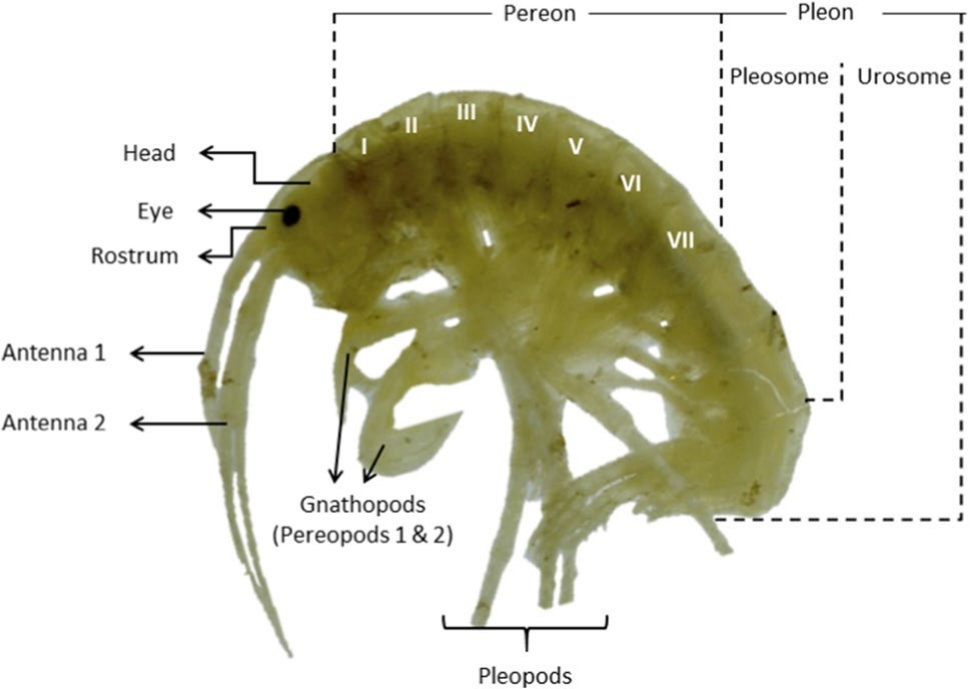
Main phenotypical features of a *Hyalella*

The automated method for counting and extracting phenotypical characteristics of *Hyalella* individuals consists of two stages: the training of the model and the extraction of phenotypical features of the individuals (Fig. 1). The model training is based on the YOLOv3’s architecture, and the extraction of characteristics from individuals was performed by using the OpenCV library (Open-Source Computer Vision) version 4. 1. 2 (Zelinsky 2009) in Python 3.7 (Rossum  2018).

### Obtention of organisms and images

*Hyalella* individuals included in this study were collected from Lake Tota, located in Boyacá, Colombia (5°32′12. 8″ N 72°55′40. 7″ W). *Hyalella* is particularly abundant in this lake and has served as a bioindicator of local water quality (Pineda-Alarcón & Cañón 2023). The habitat of *Hyalella* in this lake is associated with the aquatic plant *Egeria densa* (Planch, 1849) along the lake’s shoreline. For all macroinvertebrates samplers, ever is necessary the application of a standard protocol before counting or taxonomic determine macroinvertebrates. The sample preparation requires washing the collected material two or three times with clean water through the net, being careful to retain the sample inside the net. Afterwards, the net is inspected to remove large debris, detect organisms, and place the organisms found into the sample container. In the lab, the sample is rinsed thoroughly in a 500 µm mesh sieve to remove preservative and fine sediment, and large organic material (leaves, twigs, algal or macrophyte mats, etc.). Finally, the samples are preserved in alcohol (Rice et al. 2012; Ntislidou et al. 2021).

**Fig. 2:**
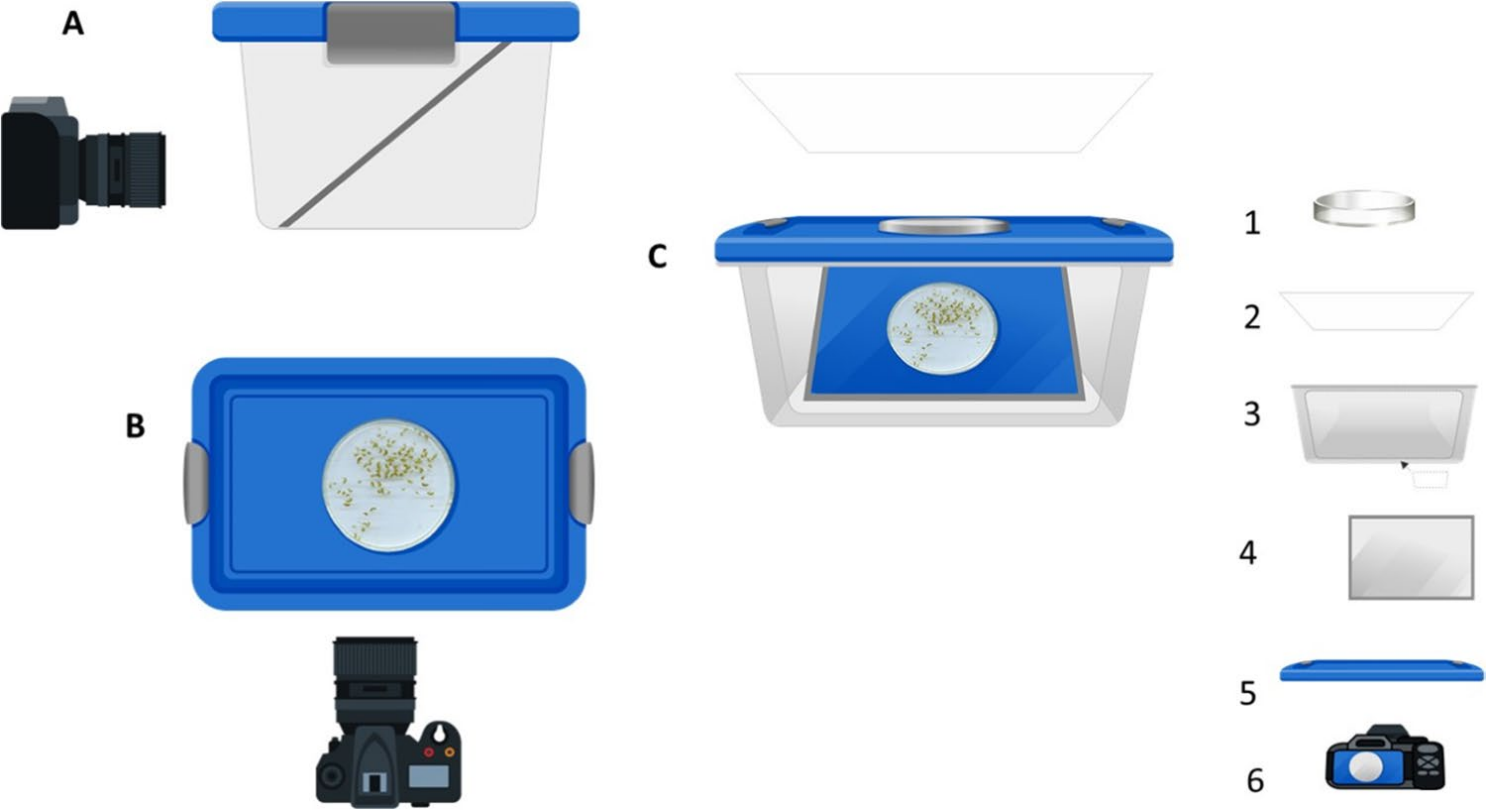
Outline and assembly elements for image capture: lateral view (**A**), upper view (**B**), and front view (**C**). Elements: Petri dish (1), sheet of paper (2), side wall of the plastic box (3), mirror (4), lid (5), camera (6)

To obtain the images as a basic input in the process of quantification and characterization of *Hyalella*s, we designed an experimental assembly that allowed the visualization of a Petri dish with the individuals fixed in 70% alcohol. In this setup, we used a transparent plastic box (25 × 15 × 20 cm) with a lid on the top in which we practiced an orifice the size of the Petri dish. We put the sample of *Hyalella*s in the Petri dish, placing the set on the orifice, and covering it with a sheet of paper to prevent the influence of external light. On the inside of the plastic box, an oblique mirror reflects on one side the contents of the background of the Petri dish, allowing the projected scene to be captured with a camera Nikon D5600, which has a 24MP CMOS sensor with no optical low-pass filter (OLPF) and the company’s latest EXPEED 4 processor. This combination offers an ISO range of 100–25,600 and 5 fps burst shooting, while the 39-point AF system can track subjects in “3D.” Other features include a fully articulating 3.2″ touchscreen LCD with 1.04 M dots, 1080/60p video, and Wi-Fi with Bluetooth for a constant connection, plus NFC for quick pairing with Android devices (Fig. 2).

To avoid sources of noise at the imaging stage, samples from the Petri dish should contain evenly distributed individuals, removing any waste element as much as possible and avoiding the overlapping of individuals or the contact with the periphery of the Petri dish and air bubbles.

### Method for counting and characterizing individuals

**Fig. 3:**
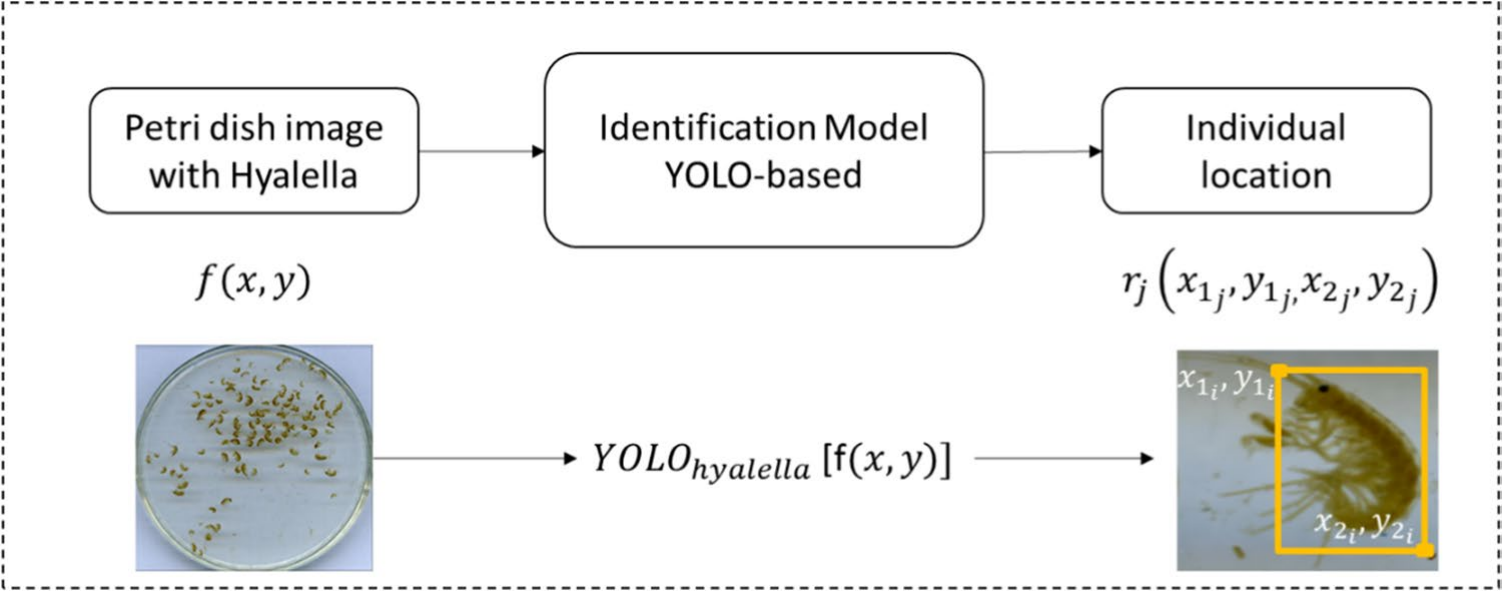
Prediction model for the *Hyalella* count created with YOLO. From an original image $f\left(x,y\right)$, the model returns the total number of individuals and the region ${r}_{j}$ occupied by each of the individuals

We based our detection method on YOLO (Redmon et al. 2016), taking an image $f\left(x,y\right)$ of a Petri dish containing individuals of the genus *Hyalella*, and returning an inventory of all regions ${r}_{j}\left({x}_{{1}_{j}},{y}_{{1}_{j},}{x}_{{2}_{j}},{y}_{{2}_{j},}\right)\in f\left(x,y\right)$ containing a *Hyalella*, indicating the organism’s body, delimited by the rectangle with a top left vertex ${x}_{{1}_{j}},{y}_{{1}_{j}}$ and bottom right vertex ${x}_{{2}_{j}},{y}_{{2}_{j}}$ for each individual *j* identified. Figure 3 summarizes the prediction model for identifying and counting *Hyalella* individuals.

#### Training of the prediction model

To train a model using the YOLO’s architecture, two types of inputs are needed: parameters configuration $\left\{{p}_{1},{p}_{2},\dots ,{p}_{n}\right\}$ and an image bank with delimited regions containing individuals to identify ${z}_{j}\left({x}_{{1}_{j}},{y}_{{1}_{j},}{x}_{{2}_{j}},{y}_{{2}_{j}}\right)$.

**Fig. 4:**
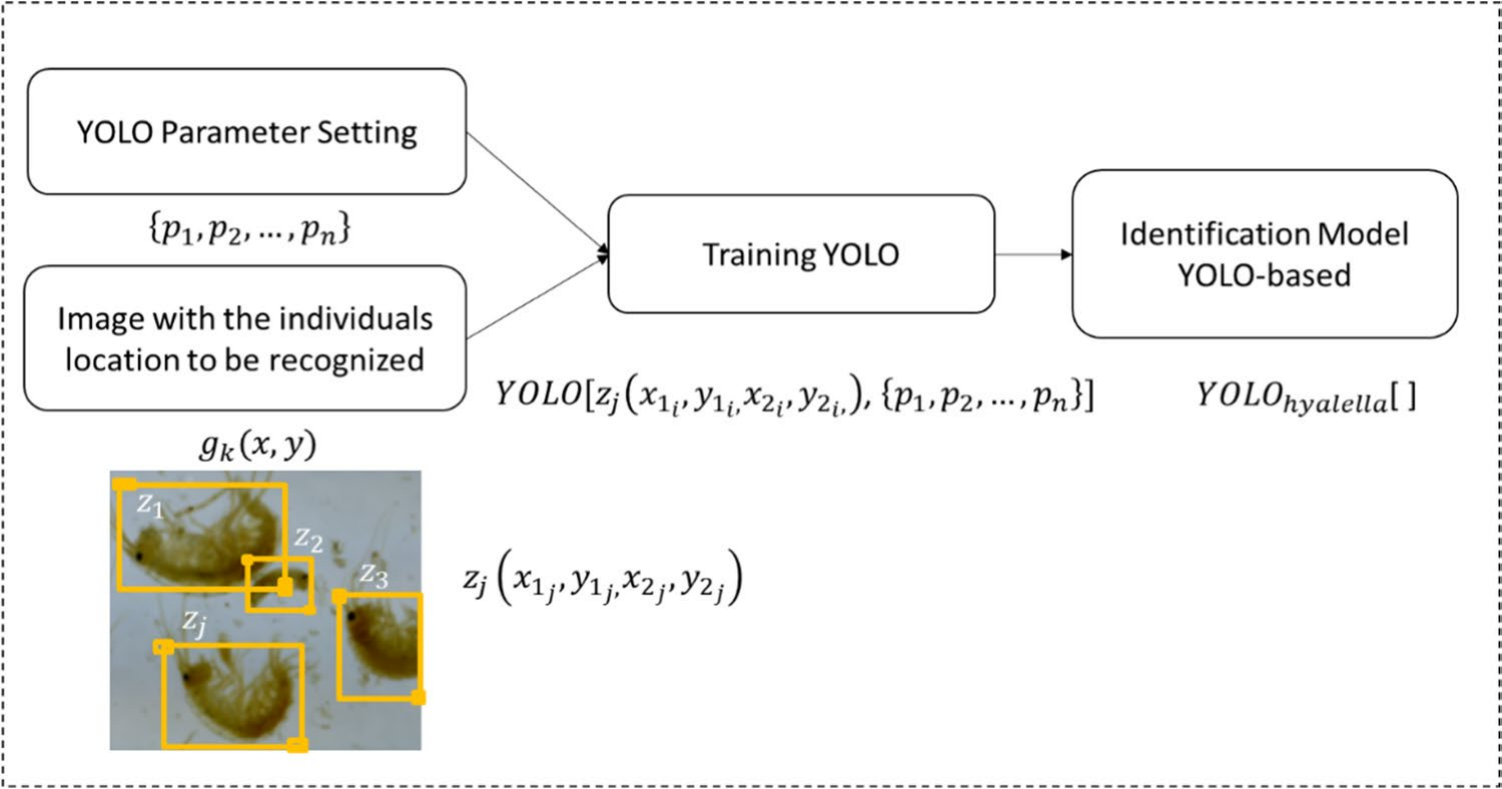
Training YOLO algorithm for the identification of *Hyalella* individuals. The parameters set $\left\{{p}_{1},{p}_{2},\dots .{p}_{n}\right\}$ and the interest regions ${z}_{j}\left({x}_{{1}_{j}},{y}_{{1}_{j},}{x}_{{2}_{j}},{y}_{{2}_{j}}\right)$ are the inputs to train the ${YOLO}_{\mathrm{Hyalella}}[ ]$ model

This study used YOLOv3 with the open-source framework darknet and the neural network structure recommended by the community in the GitHub repository, https://github.com/AlexeyAB/darknet, that allows defining parameters such as network size (i.e., width of 416 pixels and height of 416 pixels), learning rate (i.e., 0.001), number of training iterations (i.e., 10000), among others (the file with the configuration parameters used in this work is provided as supplementary material). In addition, the labeling process is based on the tool recommended in the GitHub repository https://github.com/ManivannanMurugavel/Yolo-Annotation-Tool-New-, which provides a GUI to easily select the bounding boxes and automatically generate the files with the normalized values of the bounding boxes. We used a set of portions ${g}_{k}\left(x,y\right)$ of the original image to enter the data of the individuals for training. The regions ${z}_{j}\left({x}_{{1}_{j}},{y}_{{1}_{j},}{x}_{{2}_{j}},{y}_{{2}_{j}}\right)$, containing the individuals to be used for training, were manually determined from the set of portions (Fig. 4). We applied the data augmentation technique suggested by Mikołajczyk and Grochowski (2018) to the portion ${g}_{k}\left(x,y\right)$, thus, obtaining enough individuals to allow the development of a good model.

#### Dataset augmentation for training

**Fig. 5:**
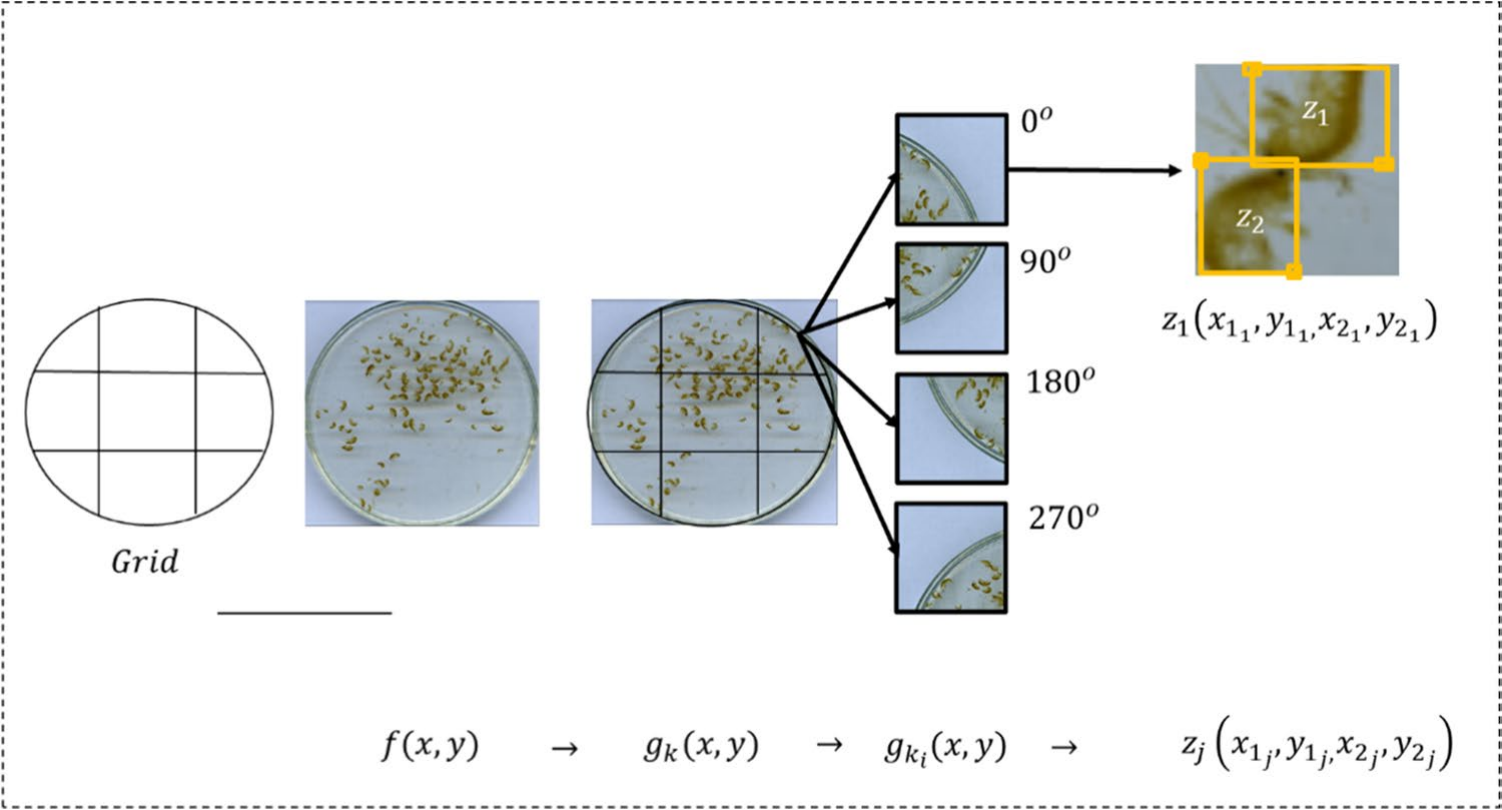
Method of database augmentation for training. Grids of 4, 6, or 9 elements divide the $f\left(x,y\right)$ image into ${g}_{k}(x,y)$ sub-images. Rotations of the sub-images create four new ${g}_{{k}_{i}}\left(x,y\right)$ sets. These sets provide the ${z}_{j}\left({x}_{{1}_{j}},{y}_{{1}_{j},}{x}_{{2}_{j}},{y}_{{2}_{j}}\right)$ regions with the individuals for training

YOLO is trained to predict the region that an object of interest occupies in a picture using a considerable number of sample images. These training images must contain multiple orientations of the object to be identified. Literature recommends no less than 1000 images for this procedure per type of individual. To achieve this training base, we used a *data-augmentation* technique (Shorten and Khoshgoftaar 2019) which consisted in dividing each image of a Petri dish $f\left(x,y\right)$ into a set of *k* sub-images ${g}_{k}(x,y)$, either of 4, 6, or 9 portions, which in turn were replicated four times by rotating them 0°, 90°, 180°, and 270° to obtain new images ${g}_{{k}_{i}}(x,y)$. With this procedure, we obtained 2781 images, from which we get the ${z}_{j}\left({x}_{{1}_{j}},{y}_{{1}_{j},}{x}_{{2}_{j}},{y}_{{2}_{j}}\right)$ regions containing the individuals to train the model (Fig. 5).

#### Extraction of morphological characteristics

**Fig. 6:**
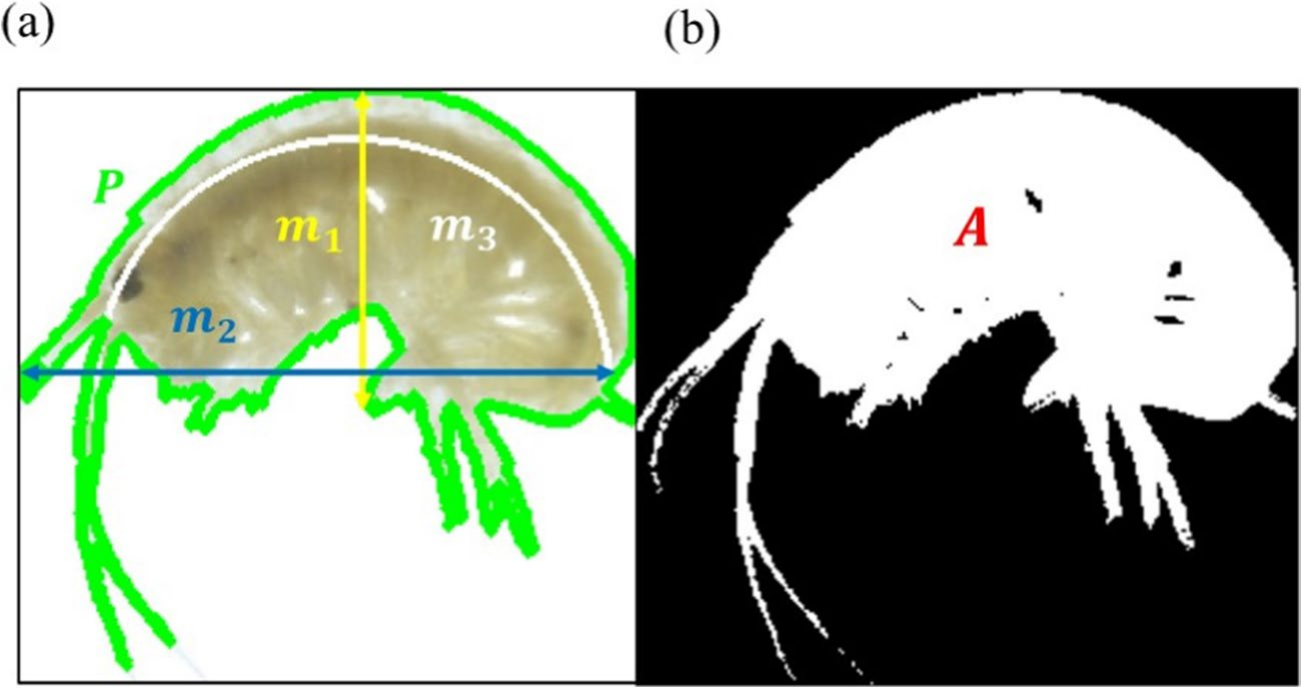
Representation of the extracted characteristics from each *Hyalella*. **a** *Hyalella*’s length (m2), maximum thickness (m1), arc length (m3), and perimeter (P); **b** *Hyalella*’s area (A)

After detecting the *Hyalella* organism in the image, we extracted the morphological characteristics listed in Table 1 for each organism framed in the regions ${r}_{\mathrm{j}}\left({x}_{{1}_{\mathrm{j}}},{y}_{{1}_{\mathrm{j}},}{x}_{{2}_{\mathrm{j}}},{y}_{{2}_{\mathrm{j}}}\right)$(Fig. 4). The length (*m2*) determines the linear measure of the individual. The arc length (*m3*) represents the total body measure along the curved shape of the individual. The maximum width (*m1*) refers to the width of the individual. The eccentricity (*e*) is a dimensionless parameter that indicates the degree of curvature of the individual. The perimeter (P) and the area (A) denote the contour and the space occupied by the organism within the Petri dish respectively (Fig. 6). The characteristics of interest were obtained using basic digital image processing techniques. Initially, the image is converted to the HSV color space (hue, saturation, value), then binarized using the Otsu method (Gonzalez and Woods 2000), continuing with the application of morphological operations to finally identify the individual’s contour. Based on this outline, the characteristics are extracted with algorithms implemented in several functions of the OpenCV library.

**Table 1:** Interest characteristics extracted from each *Hyalella* and its mathematical representation

Characteristics	Detail for the individual	Representation
Length	Maximum length in pixels of the largest side of the ellipse containing the specimen	$m1$
Maximum width	Maximum length in pixels between the ends of the specimen, perpendicular to the direction of its longest length	$m2$
Eccentricity	The ratio of the distance between the focal points of the ellipse and the length of its major axis	$e=\sqrt{1-\frac{m2}{m1}}$
Arc length	Measurement of the distance along a curve in an interval [a, b]	$m3$
Perimeter	Distance between each pair of contiguous pixels around the edge of the region	P
Area	Actual number of pixels in the region	$A=\sum_{x}\sum_{y}I(x,y)$

The values associated with the extracted characteristics were transformed from pixels to millimeters, using a scale factor based on the standardized diameter of a Petri dish, corresponding to 10 cm (Corkidi et al. 1998).

### Model validation

#### Model performance

To evaluate the performance of the model in detecting *Hyalellas*, we took as a basis the statistical values of true positives (TP-correct identification of *Hyalellas*), false positives (FP-incorrect identification of *Hyalella*s), and false negatives (FN-no detection of *Hyalellas*, when present). From these values, we constructed the confusion matrix to obtain different validation metrics such as accuracy, precision, recall, and F1-score. The training process automatically provides the results of the validation test (confusion matrix), comparing the regions generated by the model ${r}_{j}\left({x}_{{1}_{j}},{y}_{{1}_{j},}{x}_{{2}_{j}},{y}_{{2}_{j},}\right)$ with the regions provided by the expert ${z}_{\mathrm{j}}\left({x}_{{1}_{\mathrm{j}}},{y}_{{1}_{\mathrm{j}},}{x}_{{2}_{\mathrm{j}}},{y}_{{2}_{\mathrm{j}}}\right)$ that were obtained from a set of test images. The following are the formulas used for the calculation of each of these metrics.

Accuracy: percentage of *Hyalellas* correctly identified, considering the total number of *Hyalellas* delivered by the expert and the number of objects other than *Hyalellas* that were identified as such (Eq. 1).

1 $$\mathrm{Accuracy}=\frac{\mathrm{Number}\;\mathrm{of}\;\mathrm{correct}\;\mathrm{detections}}{\mathrm{Total}\;\mathrm{number}\;\mathrm{of}\;\mathrm{Hyalellas}\;+\;\mathrm{FP}}$$

Precision: number of *Hyalellas* correctly identified, considering the number of identified objects, i.e., the cases that were correctly detected plus the cases other than *Hyalellas* that were identified as such (Eq. 2).

2 $$\mathrm{Precision}=\frac{\mathrm{Number}\;\mathrm{of}\;\mathrm{correct}\;\mathrm{detections}}{\mathrm{Total}\;\mathrm{number}\;\mathrm{of}\;\mathrm{dectections}}$$

Recall: percentage of *Hyalellas* that the system can correctly identify based on the quantity supplied by the expert (Eq. 3).

3 $$\mathrm{Recall}=\frac{\mathrm{Number}\;\mathrm{of}\;\mathrm{correct}\;\mathrm{detections}}{\mathrm{Total}\;\mathrm{number}\;\mathrm{of}\;\mathrm{Hyallelas}}$$

F1-score: measurement whose purpose is to weigh the importance of accuracy and recall (Eq. 4).

4 $$\mathrm{F}1\mathrm{ score}=\frac{2\times \mathrm{Precision}\times \mathrm{recall}}{\mathrm{Presicion}+\mathrm{recall}}$$

#### Comparison between traditional counting and the model developed

To compare traditional and automated quantification methods, we counted the *Hyalellas* in 36 images using both visual inspections by a trained person in the microscope and the model developed. We recorded the time spent in seconds for each method and applied a non-parametric Mann–Whitney test to identify differences (Rencher and Schimek 1997). The results obtained were also compared with the Spearman’s correlation method.

## Results

**Fig. 7:**
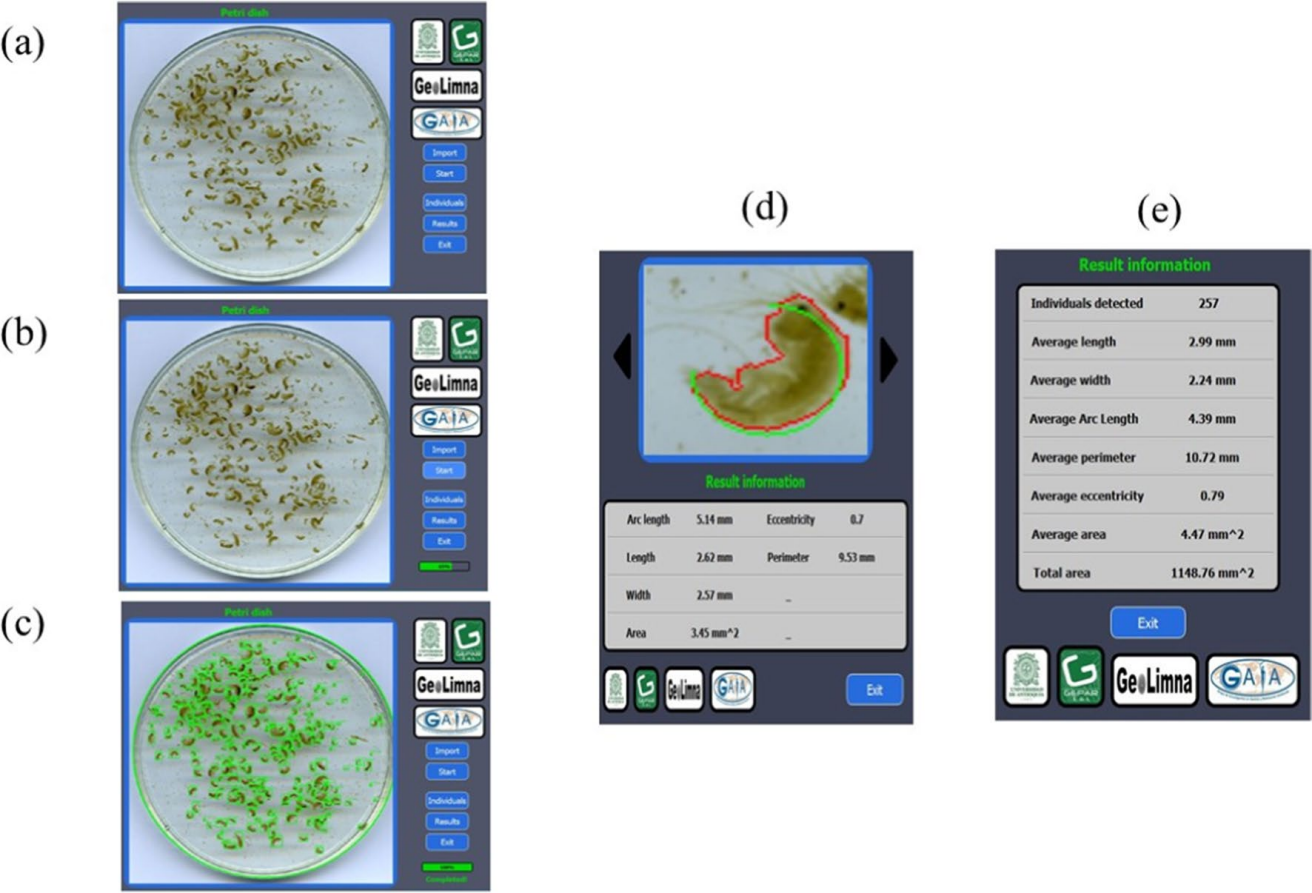
*Hyalellas* identification, counting, and body metrics extraction in its various stages: **a** importing the input image; **b** detecting and extracting characteristics; **c** visualizing detected individuals; **d** visualizing individual morphological characteristics; **e** averaging characteristics per processed image

**Fig. 8:**
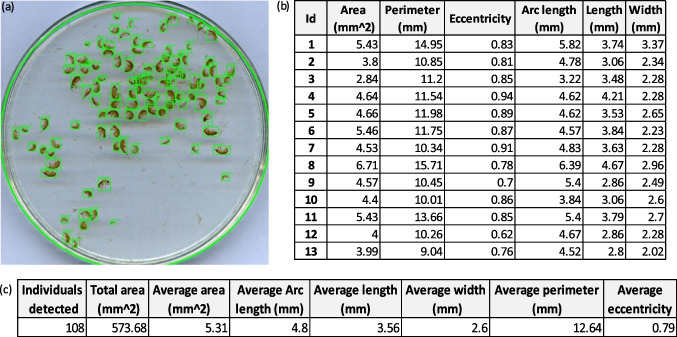
Individuals detected and spreadsheet report generated by HyACS after processing: **a** Labels of the Hyalella individuals detected. **b** Metrics per individual identified. **c** Average metrics of the individuals identified per image

Figure 7 shows the HyACS software interface and the step-by-step process of the *Hyalellas* identification, counting, and body metrics extraction. The software uses as input an image of a Petri dish containing an unknown amount of *Hyalellas* (Fig. 7a), and the process starts where the user can track the status through the progress bar displayed on the software interface (Fig. 7b). To identify and quantify the individuals in the image, HyACS relies on the model generated using YOLO, which indicates the regions where the *Hyalellas* are located as the result of the processing (Fig. 7c). Subsequently, the software extracts each of the detected *Hyalellas* as an individual image to determine their defined morphological characteristics (Fig. 7d). Finally, it is provided the number of individuals and the metric’s extracted average (Fig. 7e). The user can access the data estimated by the software through two Excel spreadsheets. The first sheet corresponds to the data per individual and the second sheet contains the average data of the processed image (Fig. 8).

### Model validation metrics

The model performance is evaluated through the metrics shown in Table 2, which correspond to the values of *accuracy, precision, recall*, *and F1-score*, highlighting both the *recall*, which indicates that the model identifies more than 90% of *Hyalellas* correctly and the *accuracy* that denotes that the model success in 90% of its predictions.

**Table 2:** Results of the metrics to evaluate the model’s performance

Accuracy	Precision	Recall	F1-score
0.92	0.80	**0.92**	0.86

Data in bold emphasis indicate the recall and accuracy because these metrics are above 90%

#### Visual counting vs. model developed

**Fig. 9:**
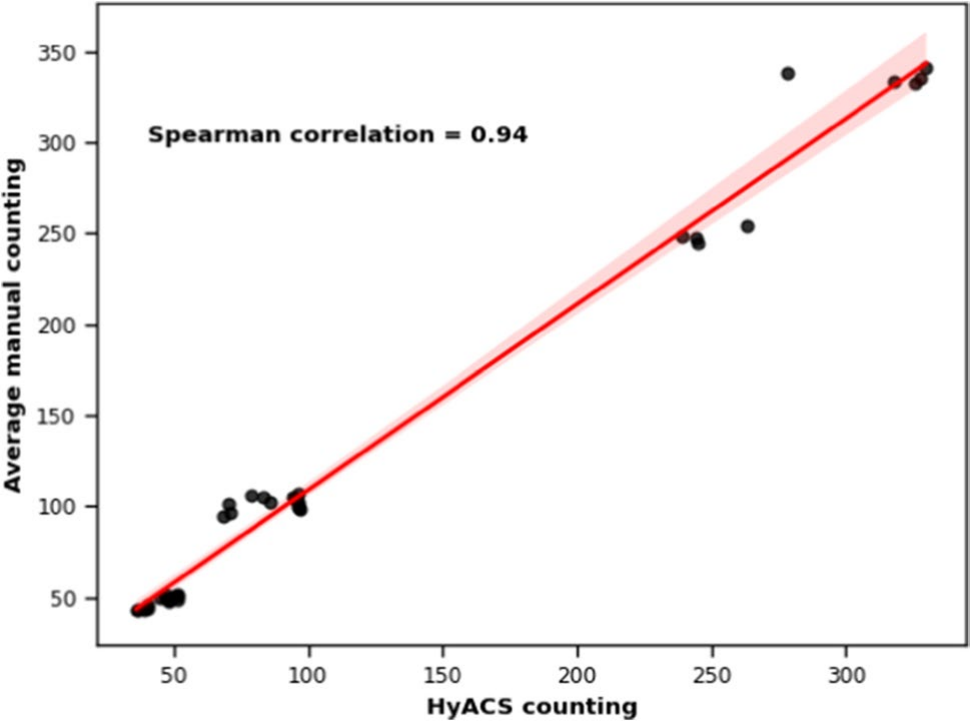
Correlation of visual counting by an expert vs. automatic counts by the model. The red line represents the best linear fit

Figure 9 shows the Spearman correlation between the number of *Hyalellas* counted by visual inspection (average manual counts from 3 different people) and the number of *Hyalellas* detected by the model. The correlation coefficient ${r}_{2} = 0.94$ indicates a high correlation degree between the counts carried out by each method.

Upon comparing the counting time of visual inspection and the model, we observed a significant difference in their distribution and dispersion. The time taken for visual counting ranged widely from 46 to 409 s, with an average of 150 s. Conversely, automatic counting demonstrated minimal variation, ranging from 22 to 58 s, with an average of 35 s. The statistical analysis indicated that there is a statistically significant difference between the two counts, with a *p*-value less than 0.05.

## Discussion

Through the correlation analysis between manual and automatic counting, we identify a positive correlation coefficient, suggesting that the model performs correctly. Likewise, we compared the time required for counting and extracting individual characteristics between the two methods and we found that the use of HyACS led to a time reduction of approximately four times. This is one of the highlights of the software, because, in similar studies, Lins et al. (2020), also achieved a fourfold reduction in time using the software (Aphid CV). Therefore, HyACS contributes efficiently to the counting and measurement of individuals of the *Hyalell*a genus.

In the validation of the model’s performance, we obtained high prediction rates with metrics above 90% for the correct identification of individuals (accuracy and recall). These results are close to those obtained in the work of Lins et al. (2020) who use a similar number of training and validation images. Meanwhile, Ding and Taylor (2016) report 93% accuracy for automatic detection of moths with a database five times larger than ours, which indicates that the greater the database the higher the accuracy.

Using HyACS, it is possible to obtain accurate body measurements and other morphological characteristics of individual organisms, which can be of biological and environmental significance (Duckworth et al. 2019). Even in studies focused on describing new species of the genus *Hyalella*, body length is often used as a distinctive measure for identifying different stages of the population from various sizes (Marrón-Becerra et al. 2020). This metric contribute in the classified as males (M), immature females (F), sexually mature females (MF), ovigerous females (OF), and juveniles (J), according to specific criteria (de Paula et al. 2021). The genus *Hyalella* exhibits a wide range of body length, varying between 1.5 and 7.2 mm in the literature (González and Watling 2003; Kühr et al. 2018). However, it is important to note that obtaining of this metric of body length is through linear length measurement that may not fully account for the curved shape of the genus or differences in curvature that may arise when organisms are fixed for analysis (Duckworth et al. 2019).

Studies, such as de Paula et al. (2021), calculate a more accurate body length, by sliding *Hyalella*’s body, measuring body segments individually and adding these measurements to obtain the total length (in millimeters). This process requires a greater investment of time and work. However, HyACS provide a more accurate and efficient body length measurement by detecting automatically the arc length along the curve of the individual. In this regard, HyACS body lengths measurements exceed the maximum length reported in the literature, indicating that traditional techniques may underestimate the body length of organisms (Duckworth et al. 2019).

Ecological studies often involve analyzing the size spectra and organization of communities, where body measurements play a crucial role (Pomeranz and Wesner 2022). Some studies use body length and maximum thickness as dispersion limiting factors among others to understand changes in *Hyalella* populations (Li et al. 2021). For instance, changes in thickness whether the organisms are thicker or thinner could indicate changes in its environment conditions (De Marchi et al. 2019). Various metrics are used in biotic communities to determine the biomass of individuals. Some studies determine the biomass through weight and length (Gualdoni et al. 2013; Stoffels et al. 2003), while others determine the weight from biomass and length to identify the communal energy contribution within an aquatic ecosystem (Rivera-Usme et al. 2015). Additionally, the relationship between body size and dry biomass can be estimated based on the measured body length to determine toxicity of fipronil and 2,4-D in *Hyalella meinernti* (da Silva Pinto et al. 2021).

Eccentricity is a measure that allows the evaluation of the curvature of the *Hyalella*. This characteristic indicates circular shapes for values close to zero and linear shapes for values close to one. This measure may be a response to various environmental conditions or a species differentiating characteristic (Marrón-Becerra et al. 2020). These characteristics can be obtained through HyACS for the *Hyalella* genus. However, depending on the research question and purpose, the software can be adjusted using tools such as OpenCV library to estimate multiple morphological measurements. In addition, measures such as area and length can be inputs to estimate biologically important metrics such as biomass or weight (Gualdoni et al. 2013; Stoffels et al. 2003).

For better results, both in counting and in measuring characteristics, it is essential to locate the organisms in a way that avoids the edges of the Petri dish. Additionally, it is important to avoid the formation of air bubbles that prevent the bottom of the box from being delimited and the overlapping of organisms.

Using a high-resolution camera that produces quality images can also facilitate and improve the counting and measuring of *Hyalella*’s characteristics. Other authors with similar models have provided this type of recommendation (Lins et al 2020), reaffirming the importance of sample preparation for the acquisition of the image to get a better performance.

The use of software such as HyACS provides a new approach to counting and measuring *Hyalella* that challenges traditional standardized methods. This method also offers the possibility of modifying the sampling protocol since images can be acquired directly in the field. As a result, organisms can be returned to their environment without the need to fix and transport the samples. This approach not only eliminates the need to stress the organisms with the traditional process but it also avoids the alteration of their shape and limbs, which can occur due to the stress of the process and result in their death (Rice et al. 2012).

## Conclusions

HyACS is a software that accurately identifies, counts, and extracts shape characteristics from *Hyalella* organisms. It provides fast and reliable results for researchers interested in analyzing data related to the ecology and management of an aquatic ecosystem.

Compared to traditional methods, HyACS offers significant advantages in terms of time spent on counting and the reliability of the results, providing a more accurate population dynamic description. This opens up new possibilities for improving sample processing of the *Hyalella* genus in laboratory analysis and sampling protocols of individuals in the field. HyACS provides more reliable results on population dynamics and realistic view of the ecosystem.

HyACS can be applied in research related with the demographic, ecological, and toxicological implications of *Hyalellas*, where precise measurements of size and abundance are required. The software would be advantageous to obtain body measurements that consider the curvature of the body as a determining factor in population morphology (Duckworth et al. 2019). These results about metrics brought by HyACS are fast, automatic, and reliable.

The integration of machine learning techniques in fields like biology and ecology is the result of joint efforts from various disciplines. It is therefore important to make progress on methodologies that facilitate the collection of data in populations that are difficult to count or measure and that have been standardized for years using methods that do not consider species-inherent variations, such as the *Hyalella* curvature. HyACS is a pioneering software that uses convolution neural network YOLO to identify objects and employs basic image processing techniques to determine relevant morphological characteristics in the genus *Hyalella*. Its introduction marks a significant advancement in the counting and quantification of characteristics in this genus. We hope that future studies in this area will use HyACS to enhance the quality and reliability of their results.

## Data Availability

The datasets generated and/or analyzed during the current study are available from the corresponding author on reasonable request.
